# Enhancing Junior Doctors’ Preparedness and Satisfaction in Trauma and Orthopaedics: A Quality Improvement Project With the Development of a Comprehensive Guidebook

**DOI:** 10.7759/cureus.70061

**Published:** 2024-09-23

**Authors:** Ghulam Dastagir Faisal Mohammed, Zubair Younis, Jebran Amin, Zaina Mansoor, Leonie Lingnau, Edwin P Jesudason

**Affiliations:** 1 Trauma and Orthopaedics, Ysbyty Gwynedd, Bangor, GBR; 2 Trauma and Orthopaedics, Royal Shrewsbury Hospital, Shrewsbury, GBR; 3 Trauma and Orthopaedics, The Robert Jones and Agnes Hunt Orthopaedic Hospital, Oswestry, GBR; 4 Cardiology, Ysbyty Gwynedd, Bangor, GBR; 5 Trauma and Orthopaedics, Charité – Universitätsmedizin, Berlin, DEU

**Keywords:** guidebook development, medical induction, night shift confidence, quality improvement project (qip), resident satisfaction

## Abstract

Background: Junior doctors often feel underprepared for their trauma and orthopaedics (T&O) rotation due to limited exposure during medical school and inadequate support. This project aimed to enhance junior doctors' preparedness and satisfaction during their T&O rotation by developing a comprehensive guidebook that addresses key orthopaedic knowledge and logistical challenges.

Methods: A quality improvement project (QIP) was conducted at Ysbyty Gwynedd Hospital. Initial surveys identified factors contributing to poor experiences during the trauma and orthopaedics rotation, including limited knowledge of orthopaedic emergencies and a lack of useful reference resources. A guidebook was developed and refined through multiple plan-do-study-act (PDSA) cycles. The guidebook covered topics such as orthopaedic emergencies, common injuries, referral pathways, and hospital logistics, presented in an accessible flowchart format.

Results: The primary objective of achieving 75% satisfaction among junior doctors was successfully met, with satisfaction increasing from four (40%) to eight (80%) doctors in the most recent survey. Secondary outcomes included a marked improvement in the understanding of quality improvement projects, rising from three (30%) to eight (80%) doctors. Orthopaedic knowledge also saw a significant enhancement, increasing from four (40%) to nine (90%) doctors. Confidence in handling night on-call duties improved dramatically, with all 10 doctors (100%) reporting increased confidence, compared to four (40%) doctors initially. Additionally, seven doctors (70%) expressed a greater interest in pursuing a career in orthopaedic surgery.

Conclusion: The comprehensive guidebook significantly improved junior doctors’ preparedness and satisfaction during their T&O rotation. While the guidebook is a valuable resource, ongoing mentorship and hands-on experience remain essential for long-term success. Replication of this project across other departments and hospitals is recommended to assess its broader applicability and impact.

## Introduction

The General Medical Council’s 'Tomorrow’s Doctors’ document states that ‘students must be properly prepared for their first day as a Pre-Registration House Officer' [1]. However, in a 2005 national survey, 41.5% of new doctors reported feeling inadequately prepared, with respondents noting that there was 'not enough emphasis on real-life situations' and 'insufficient time spent shadowing' [2]. Experiencing feelings of being overwhelmed and underprepared is thought to heighten stress levels in junior doctors, potentially diminishing their quality of life [3]. Newly qualified Foundation year one doctors starting work in the UK generally participate in a corporate induction, which includes mandatory training such as fire safety, manual handling, basic life support, and antibiotic guidelines, with the content varying according to local hospital policies [4]. However, this induction does not provide specific training to prepare them for the practical realities of the workplace [4]. It has subsequently become clear that new doctors entering any rotation require targeted induction specific to that particular specialty.

Many junior doctors often feel underprepared for their trauma and orthopaedics (T&O) rotation. This is largely due to insufficient exposure to the specialty during medical school, combined with inadequate support both at the start and throughout the rotation. The time it takes for residents to acclimate to the different protocols, expectations, and environments of each unique rotation adds to the challenge [5]. These factors can make it difficult to enjoy the work and may discourage doctors from pursuing T&O as a career. Night shifts are particularly stressful, with the registrar off-site and a single junior doctor responsible for managing new referrals and orthopaedic inpatients. To address these challenges, we developed a T&O guidebook designed to help juniors integrate into the department and provide a reference for the basic management of key orthopaedic conditions they may encounter while on-call.

When doctors have clinical questions, they rely on resources that are familiar, available, and of high yield [6]. We hypothesized that developing a handheld guidebook could become a familiar, accessible, and highly effective resource for core information. While the guidebook would have a nominal financial impact, its creation and maintenance would be a major investment in time [5].

## Materials and methods

**Figure 1:**
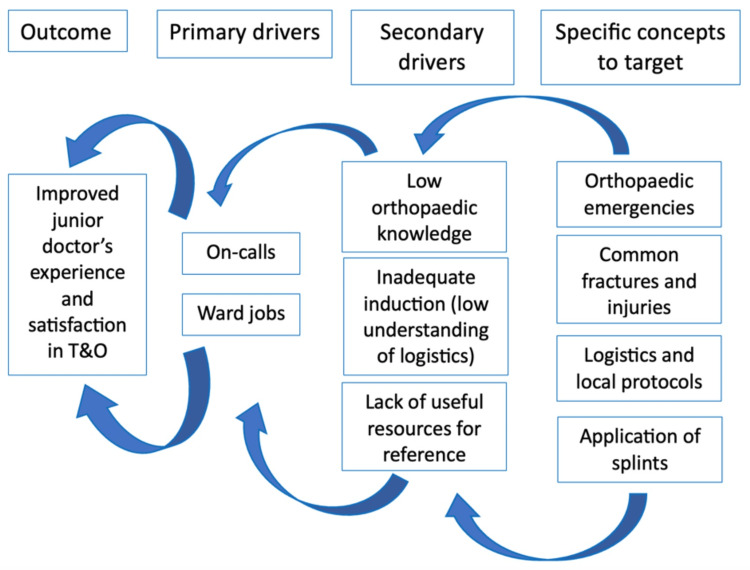
Driver diagram created during the initial quality improvement project (QIP) to determine the best strategies for enhancing the T&O experience for junior doctors, identifying the root causes of low satisfaction Image credits: First author

Step 1: An initial departmental quality improvement project (QIP) identified several key factors contributing to the poor experience of junior doctors in trauma and orthopaedics (T&O) at Ysbyty Gwynedd Hospital. The results of the initial quality improvement project (QIP) among foundation doctors indicated that the trauma and orthopaedics (T&O) rotation was particularly challenging, primarily due to the demands of on-call duties and ward responsibilities. The secondary factors contributing to these difficulties included limited orthopaedic knowledge, inadequate understanding of logistics, and a lack of useful reference resources. The areas identified under orthopaedic knowledge included familiarity with orthopaedic emergencies, working knowledge of common fractures and injuries, understanding of splints, and knowledge of local logistics and layout. These findings are summarized in the driver diagram shown below (Figure 1).

Step 2: After identifying the underlying causes, we set a clear objective using the SMART framework, which ensures that goals are specific, measurable, achievable, relevant, and time-bound (SMART). A team was formed that included key stakeholders, such as junior doctors, registrars, and consultants, to ensure a collaborative approach. Our SMART objective was to achieve at least 75% satisfaction, as measured by a single survey conducted at the end of rotation (four months).

Step 3: A baseline measurement was obtained through a survey administered to all junior doctors at Foundation or equivalent level in the trauma & orthopaedics (T&O) department. The survey, conducted via Microsoft Forms (Redmond, USA), gathered responses from outgoing junior doctors to ensure comprehensive feedback.

Step 4: PDSA Cycle 1: We introduced a guidebook as part of the induction package. Version one of the guidebook included three sections: orthopaedic emergencies, common injuries and splinting, and referral pathways within the hospital. Based on the comments received, we revised the guidebook, adding more detailed sections and incorporating flowcharts.

PDSA Cycle 2: The updated version of the guidebook was introduced and is now well-structured into distinct sections. A section on logistics, guidelines, and local protocols was added. All conditions were presented in a flowchart format, which is easy to read and had all practical points.

Step 5: Sustainability: To ensure the sustainability of the guidebook, we have made the survey a mandatory part of the process for each outgoing batch of junior doctors. This initiative also involves the incoming junior doctors going through the guidebook as part of their induction and taking up this rolling QIP project to update it. This approach not only keeps the guidebook up-to-date but also allows the junior doctors to learn and participate in the steps of a quality improvement project, helping their understanding of QIP processes.

The guidebook has now undergone five PDSA cycles. This study was started in October 2022, with the most recent survey done in February 2024 at Ysbyty Gwynedd Hospital, Bangor.

Contents of the guidebook

The guidebook is organized under the following headings:

*Introduction to the Department*: Overview of departmental procedures, including how trauma meetings are conducted, the structure of on-calls, and the process for requesting leave.

*Orthopaedic Emergencies*: Essential information on managing urgent orthopaedic cases.

*Upper Limb Injuries*: Guidance on the assessment and treatment of common upper limb injuries.

*Lower Limb Injuries*: Detailed information on diagnosing and managing lower limb injuries.

*Soft Tissue Injuries*: Overview of common soft tissue injuries and their treatment.

*Paediatric Fractures*: Special considerations and management of fractures in children.

*Common Plasters and Immobilisation Techniques*: Instructions on applying plasters and other immobilisation techniques.

**Figure 2:**
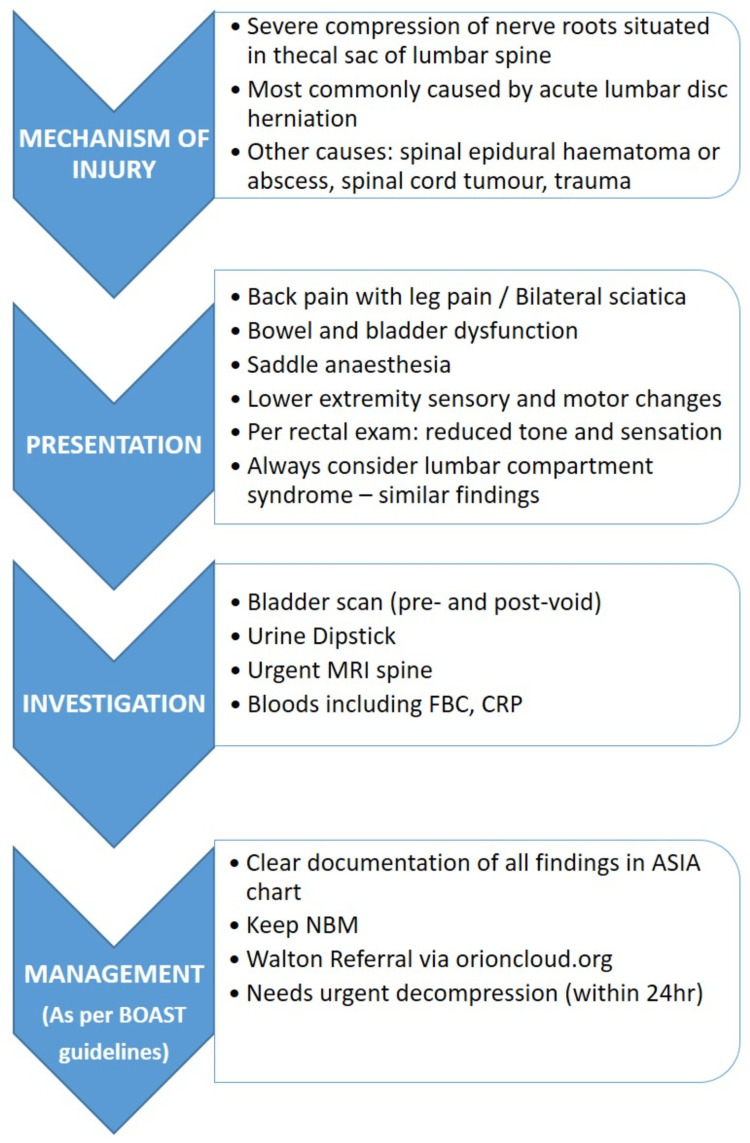
Overview of the diagnosis and management for cauda equina syndrome Image credits: First author

The entire guidebook is structured as a flowchart for each condition, providing precise information and clear steps on what actions to take for effective management (Figure 2).

## Results

**Figure 3:**
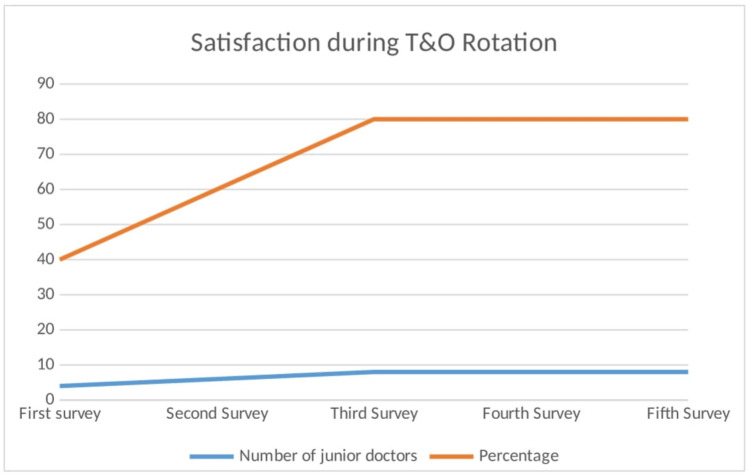
Graph depicting satisfaction during T&O rotation Image credits: Second author

The primary objective to achieve at least 75% satisfaction was successfully met, with satisfaction increasing from four (40%) to eight (80%) doctors in the most recent survey (Figure 3). In addition, all 10 (100%) junior doctors in the most recent survey felt more confident on their first night on-call shift.

Secondary objectives

**Figure 4:**
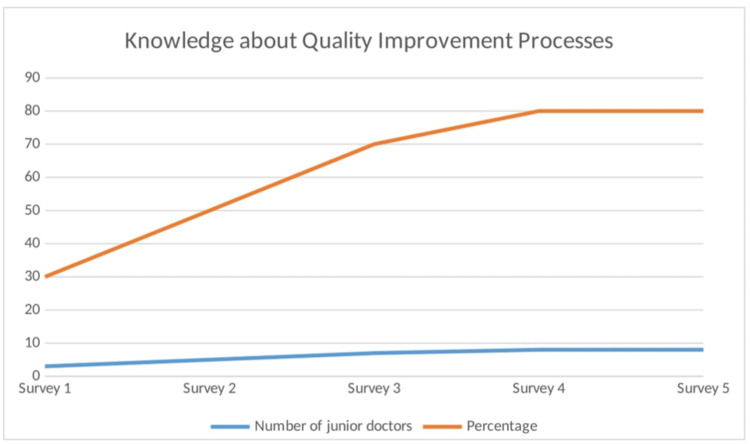
Graph depicting knowledge among junior doctors about the quality improvement process Image credits: Second author

*Learning Quality Improvement Processes*: The project has provided junior doctors with valuable experience in the steps of a quality improvement project (QIP), enhancing their understanding and skills in this essential aspect of medical practice. Over the past two years, this rolling QIP has been repeated through five cycles, involving a total of 10 junior doctors in each cycle. Following their participation, eight (80%) junior doctors reported having gained more knowledge about performing a QIP (Figure 4). Each doctor has had the opportunity to actively participate in and learn the processes of performing a QIP, further enhancing these skills in their professional development.

**Figure 5:**
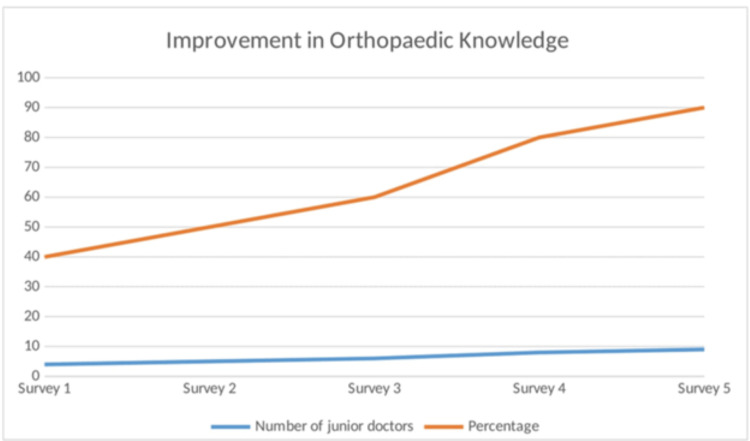
Graph depicting orthopaedic knowledge Image credits: Second author

*Enhanced Orthopaedic Knowledge:* By the end of the rotation, there has been a notable improvement in the orthopaedic knowledge of junior doctors, equipping them with a stronger foundation in managing orthopaedic cases. The most recent survey revealed that nine junior doctors (90%) felt that their knowledge had significantly improved (Figure 5).

**Figure 6:**
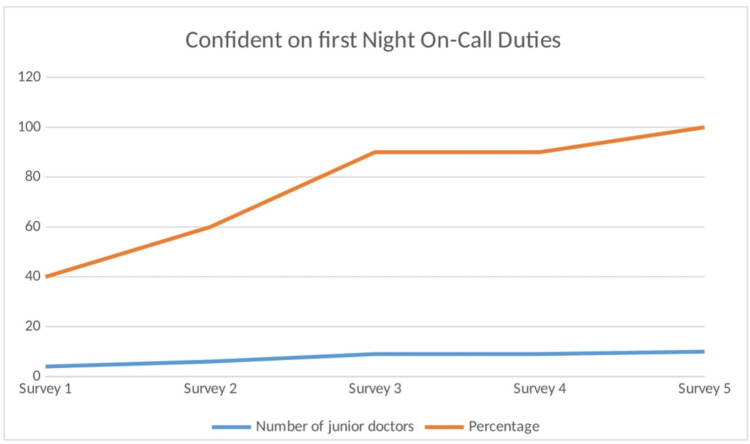
Graph depicting confidence on first night shifts Image credits: Second author

*Increased Confidence in Night On-Call Duties*: The guidebook has contributed to greater confidence among junior doctors when handling night on-call duties, a critical component of their rotation that was previously identified as a source of stress. Ten junior doctors (100%) reported being more confident about their first night on-call shift (Figure 6).

**Figure 7:**
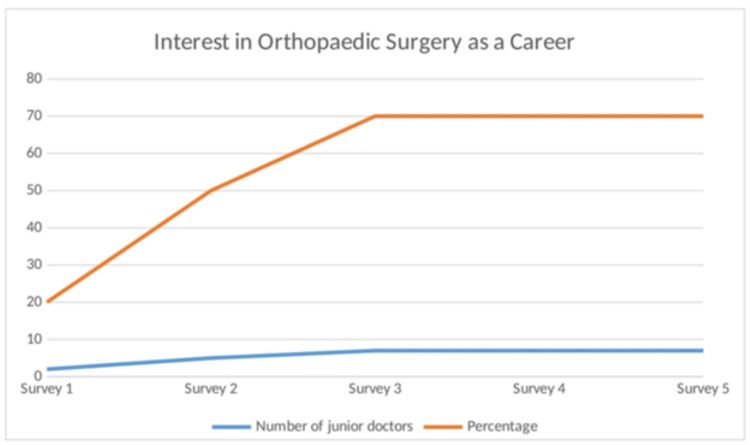
Graph depicting interest in orthopaedic surgery as a career among junior doctors Image credits: Second author

*Heightened Interest in Orthopaedic Surgery as a Career:* The improved experience and confidence gained through this initiative has sparked a growing interest among junior doctors in pursuing orthopaedic surgery as a future career, indicating the broader impact of this targeted intervention. Seven junior doctors (70%) indicated that their interest in pursuing a career in orthopaedic surgery increased as a result of participating in this project (Figure 7).

The results of the initial and most recent survey demonstrate significant improvements in satisfaction, knowledge, confidence, and career interest among junior doctors after implementing the guidebook (Table 1).

**Table 1:** Comparison of survey results on orthopaedic rotation outcomes: initial vs. recent assessments N: Number of junior doctors in each survey

Primary and secondary objectives	Initial Survey Results	Most Recent Survey Results
Satisfaction during T&O rotation in N (%)	4 (40)	8 (80)
Knowledge about Quality Improvement Processes in N (%)	3 (30)	8 (80)
Improvement in Orthopaedic Knowledge in N (%)	4 (40)	9 (90)
Confident on first Night On-Call Duties in N (%)	4 (40)	10 (100)
Interest in Orthopaedic Surgery as a Career in N (%)	2 (20)	7 (70)

## Discussion

The primary objective of achieving at least 75% satisfaction among junior doctors during their trauma and orthopaedics (T&O) rotation was successfully met, with satisfaction rising from four (40%) to eight (80%) doctors. The guidebook played a pivotal role in this improvement by offering clear and structured guidance, helping junior doctors navigate the complexities of the rotation more efficiently. Literature supports the effectiveness of structured resources in enhancing trainee satisfaction, as well-organized induction materials can significantly reduce anxiety and facilitate smoother transitions [5]. However, some authors suggest that satisfaction is multifaceted and influenced by other factors, such as mentorship and team dynamics [7]. While the guidebook clearly contributed to improved satisfaction, supportive supervision, and hands-on learning are equally important.

A key secondary objective was the improved understanding of quality improvement projects (QIPs) among junior doctors, with reported knowledge increasing from three (30%) to eight (80%) doctors. This improvement can be attributed to the direct experience of participating in QIP processes, which provided valuable hands-on learning. QIPs are essential in medical practice, as they foster critical thinking and promote continuous improvements in healthcare. Engaging in QIP projects enhances understanding, particularly when supported by efforts to increase awareness, provide mentorship, offer education on study design and implementation, and allocate resources such as books, funding, and dedicated project time [8].

The guidebook also significantly boosted orthopaedic knowledge among junior doctors, with scores increasing from four (40%) to nine (90%) doctors. This improvement is attributed to the guidebook’s clear and practical guidelines, developed by the team with a strong understanding of hospital logistics and policies. Peer-created resources like this can substantially enhance junior doctors’ knowledge and their ability to apply it effectively in real-time situations [5,9]. However, literature suggests that while a written guidebook is beneficial, it should complement rather than replace personal interactions with faculty advisers [7]. Therefore, the guidebook builds a strong foundation of knowledge but continued learning through hands-on experience remains crucial.

One of the most significant outcomes was the increase in confidence during first-night on-call duties, which rose from four (40%) to 10 (100%) doctors. This improvement aligns with findings from a study by Abukhder et al., which similarly demonstrated that structured induction materials and case-based discussions can enhance confidence [10]. This boost in confidence is crucial, as night shifts present unique challenges that can be particularly intimidating for new doctors. The guidebook played a pivotal role by providing specific protocols and guidelines for managing urgent cases, thereby reducing anxiety and empowering junior doctors to handle emergencies. Senior house officers are more comfortable in their clinical practice when they clearly understand clinical presentations, know when to escalate issues, and are familiar with the basic procedural and assessment skills expected of them [11]. This is vital not only for enhancing junior doctor confidence but also for ensuring patient safety [10]. On-call residents often experience high levels of stress and fatigue, with lower satisfaction and energy levels [12]. While the guidebook significantly boosts confidence, ongoing real-time support from senior colleagues and hands-on experience are crucial for sustaining confidence in high-pressure situations.

Thomson et al. recommended that departmental inductions for Foundation Year 1 doctors' should follow a standardised format where the content of the induction is monitored; they also recommended involving Foundation Year 1 doctors in determining the content of the induction [13]. We implemented these recommendations across all five QIP cycles by basing our interventions on feedback from outgoing junior doctors. Outgoing doctors are best positioned to identify the requirements for optimal performance in their roles, making their feedback highly relevant for guiding new doctors [10].

Lastly, the guidebook sparked a notable increase in interest in pursuing orthopaedic surgery as a career, with interest levels rising from two (20%) to seven (70%) doctors. This suggests that the guidebook not only enhanced the learning experience but also positively influenced career aspirations. Literature supports the notion that early positive experiences in specialty rotations can have a significant impact on career decisions [14]. However, it is important to recognize that career choices are influenced by multiple factors, including mentorship, exposure to diverse cases, and long-term career satisfaction, which cannot be fully addressed by a guidebook alone.

The study presents several limitations that may influence the generalizability and overall impact of its findings. Being conducted at a single hospital with a small sample size, the results may not be applicable to institutions with different structures or resources. The reliance on self-reported data also introduces a potential bias, as participants may have overstated their progress due to an awareness of the study's objectives. Additionally, the effectiveness of the guidebook is contingent on its consistent integration into the induction process, and variations in its implementation could affect the outcomes. While the guidebook serves as a valuable resource, it cannot substitute the essential role of ongoing mentorship and hands-on clinical experience, which remain critical for the sustained growth and confidence of junior doctors.

## Conclusions

This quality improvement project effectively addressed the challenges faced by junior doctors during their trauma and orthopaedics (T&O) rotation through the development of a comprehensive guidebook. Although it is difficult to fully prepare junior doctors for the complexities of a specialized rotation, this initiative demonstrated the value of a structured, multi-faceted approach to enhance both confidence and performance. The guidebook played a pivotal role in improving satisfaction by reducing stress, providing essential guidance, and enhancing both understanding of quality improvement projects and confidence in handling night on-call duties. Additionally, the initiative fostered a heightened interest in orthopaedic surgery as a career, highlighting the broader benefits of a well-structured induction process. However, the success of this guidebook underscores the importance of complementing such tools with ongoing mentorship and hands-on learning to ensure junior doctors are fully equipped to deliver high-quality patient care.

## Appendices

The study has only included 10 doctors, as there were only 10 junior doctors in a specified period who were getting replaced by a new set of junior doctors after their rotation ended.

We are happy to share a copy of the guidebook with the readers who are interested in going through it. 

## References

[1] (2003). General Medical Council Tomorrow's doctors Recommendations on undergraduate medical education London. London: General Medical Council.

[2] Cave J, Goldacre M, Lambert T, Woolf K, Jones A, Dacre J (2007). Newly qualified doctors' views about whether their medical school had trained them well: questionnaire surveys. BMC Med Educ.

[3] Paice E, Rutter H, Wetherell M, Winder B, McManus IC (2002). Stressful incidents, stress and coping strategies in the pre-registration house officer year. Med Educ.

[4] Blencowe NS, Van Hamel C, Bethune R, Aspinall R (2015). 'From scared to prepared': targeted structured induction training during the transition from medical school to foundation doctor. Perspect Med Educ.

[5] Ortiz M, Ottolini M, Agrawal D (2016). Written and online residency guidebook to improve resident efficiency and knowledge of best patient care practices. MedEdPORTAL.

[6] Thompson ML (1997). Characteristics of information resources preferred by primary care physicians. Bull Med Libr Assoc.

[7] Duff P (1995). Development of a guidebook for senior students applying for residency training in obstetrics and gynecology. Obstet Gynecol.

[8] Choudhery S, Richter M, Anene A, Xi Y, Browning T, Chason D, Morriss MC (2014). Practice quality improvement during residency: where do we stand and where can we improve?. Acad Radiol.

[9] Bedross A, Siraw BB, Alkhidir A (2024). The impact of an intern's clinical guidebook on easing the transition of new interns into the United States healthcare system. Cureus.

[10] Abukhder M, Ismail E, Dobbs T (2024). Improving burns and plastic surgery induction programmes: a departmental quality improvement project. Cureus.

[11] Rotella JA, Yu W, Ferguson J, Jones D (2014). Factors influencing escalation of care by junior medical officers. Anaesth Intensive Care.

[12] Liu CC, Wissow LS (2008). Residents who stay late at hospital and how they perform the following day. Med Educ.

[13] Thomson H, Collins J, Baker P (2014). Effective foundation trainee local inductions: room for improvement?. Clin Teach.

[14] Win Kyaw M, Cheng HC, Obermair H, Woods C, Perry C, de Costa C (2023). Australian medical students' and junior doctors' perceptions of gender discrepancies in obstetrics and gynaecology. Aust N Z J Obstet Gynaecol.

